# Supplementary material for: Cytoplasmic Interactions between the Glucocorticoid Receptor and HDAC2 Regulate Osteocalcin Expression in VPA-Treated MSCs
Source: Cells. 2019 Mar 5;8(3):217. doi: 10.3390/cells8030217 (PMC6468918; doi:10.3390/cells8030217)
Supplement: Supplementary file 1 [file cells-08-00217-s001.pdf]

Supplementary Material

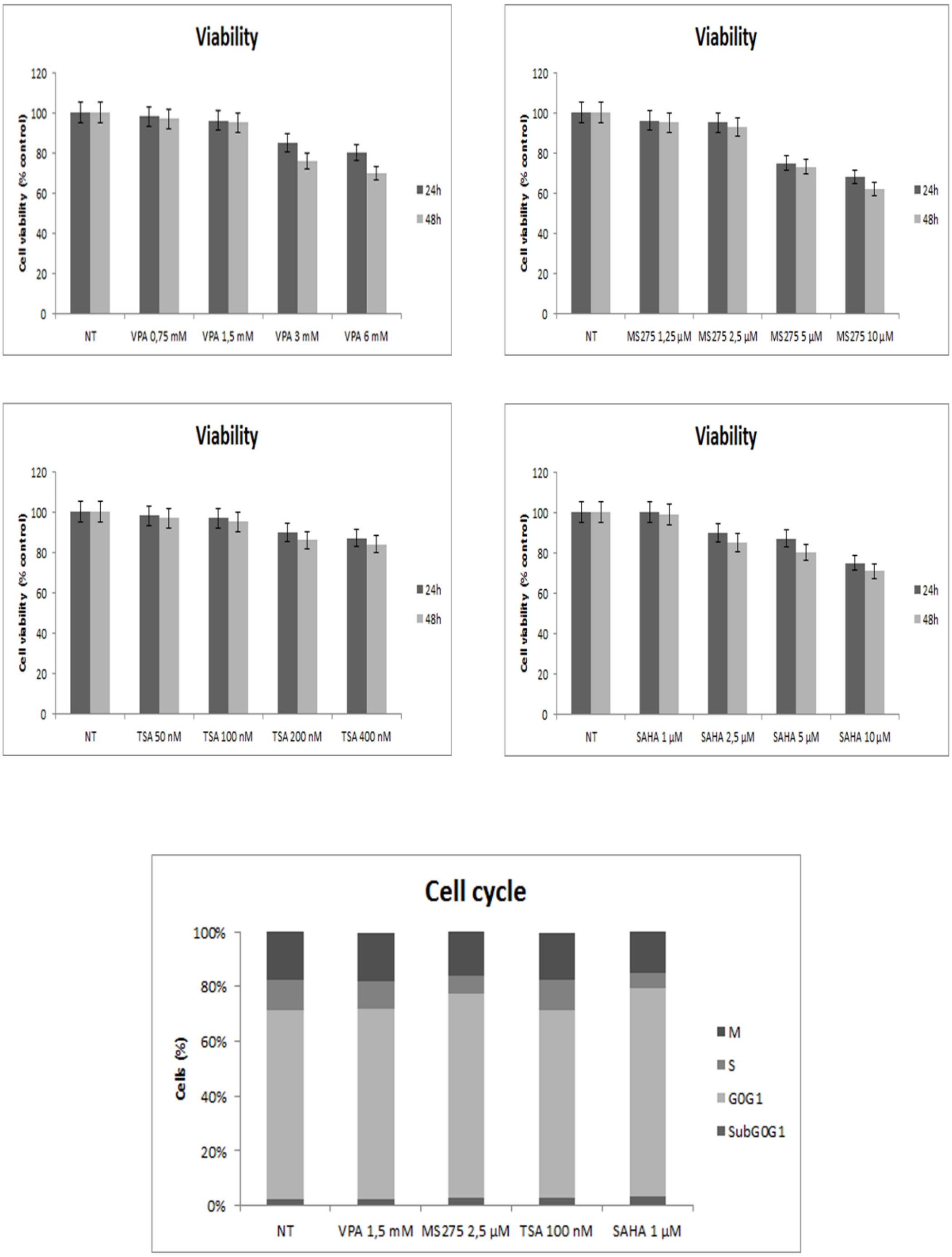

Figure 1. Viability and cell cycle analisis. Cell viability after treatment with different concentration of HDACi for 24h and 48h and cell cycle analysis of DPSCs treated with HDACi at concentrations that result nontoxic by MTT assay.

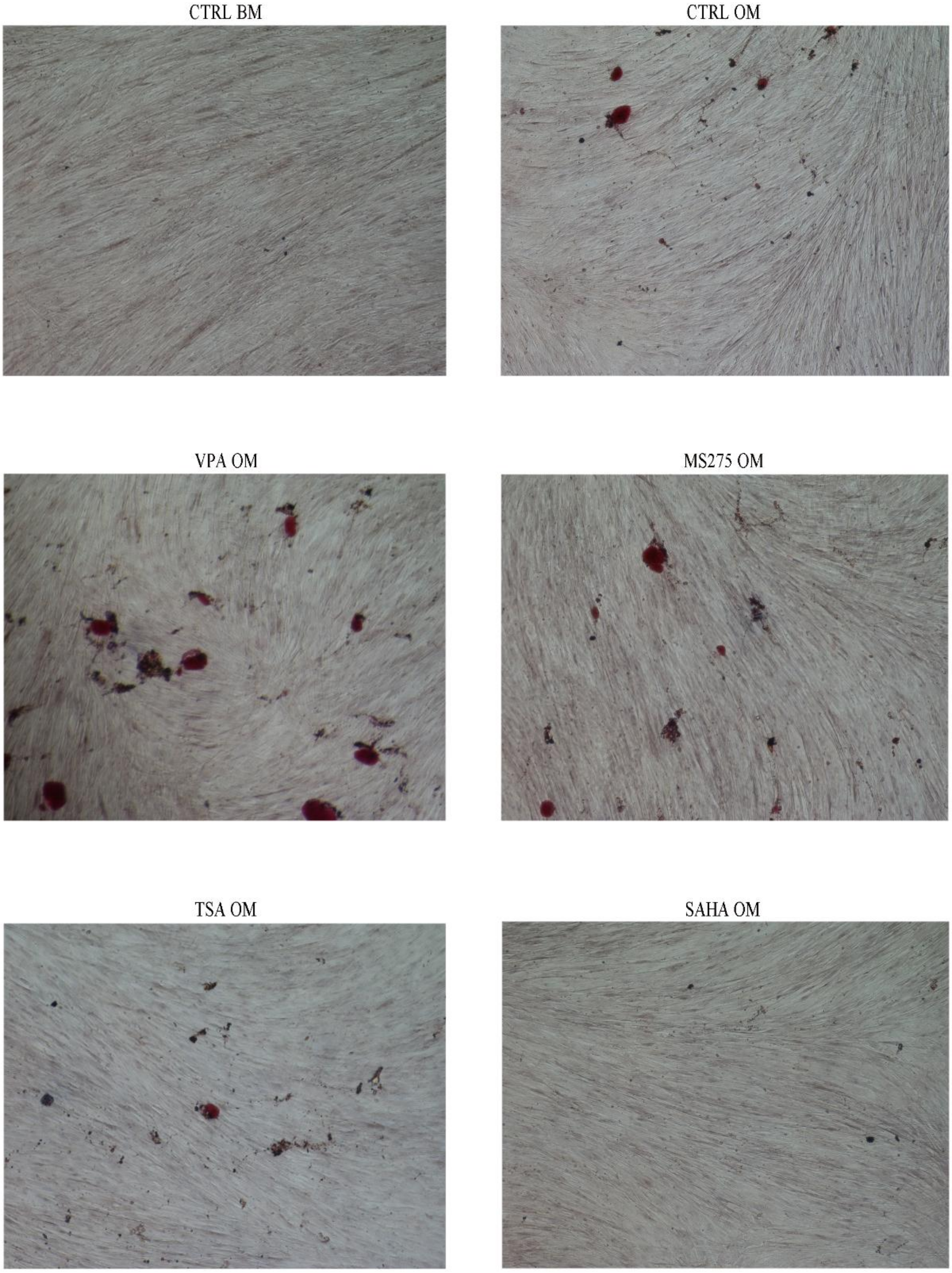

Figure 2. In vitro Alizarin red staining. Alizarin red staining of DPSCs treated for 48 h with HDACi and induced to differentiate with osteogenic medium for 21 days. It is possible to observe many nuclei of calcification in cells treated with VPA respect to control and cells treated with the other HDACi.

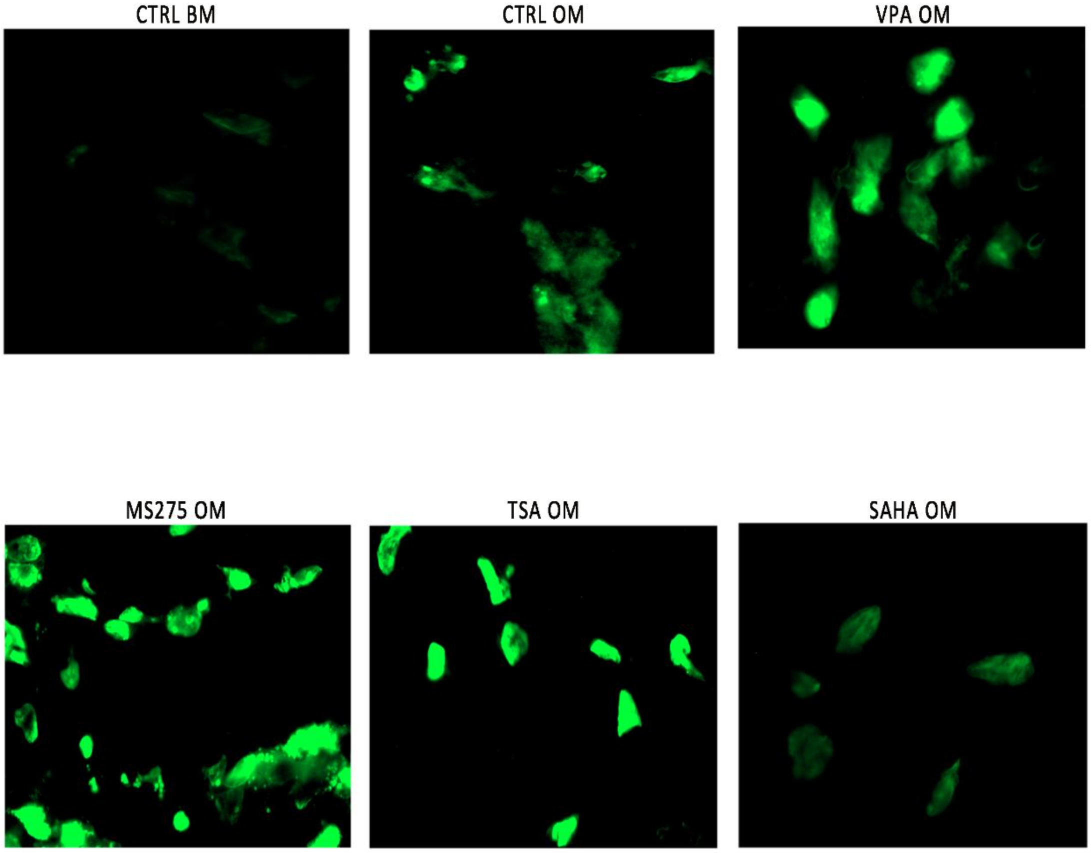

Figure 3. Immunofluorescence staining for Human nuclear antigen. To demonstrate the presence of human cells in the explants an immunofluorescence staining for the specific human nuclear antigen was performed. Magnification 100×.

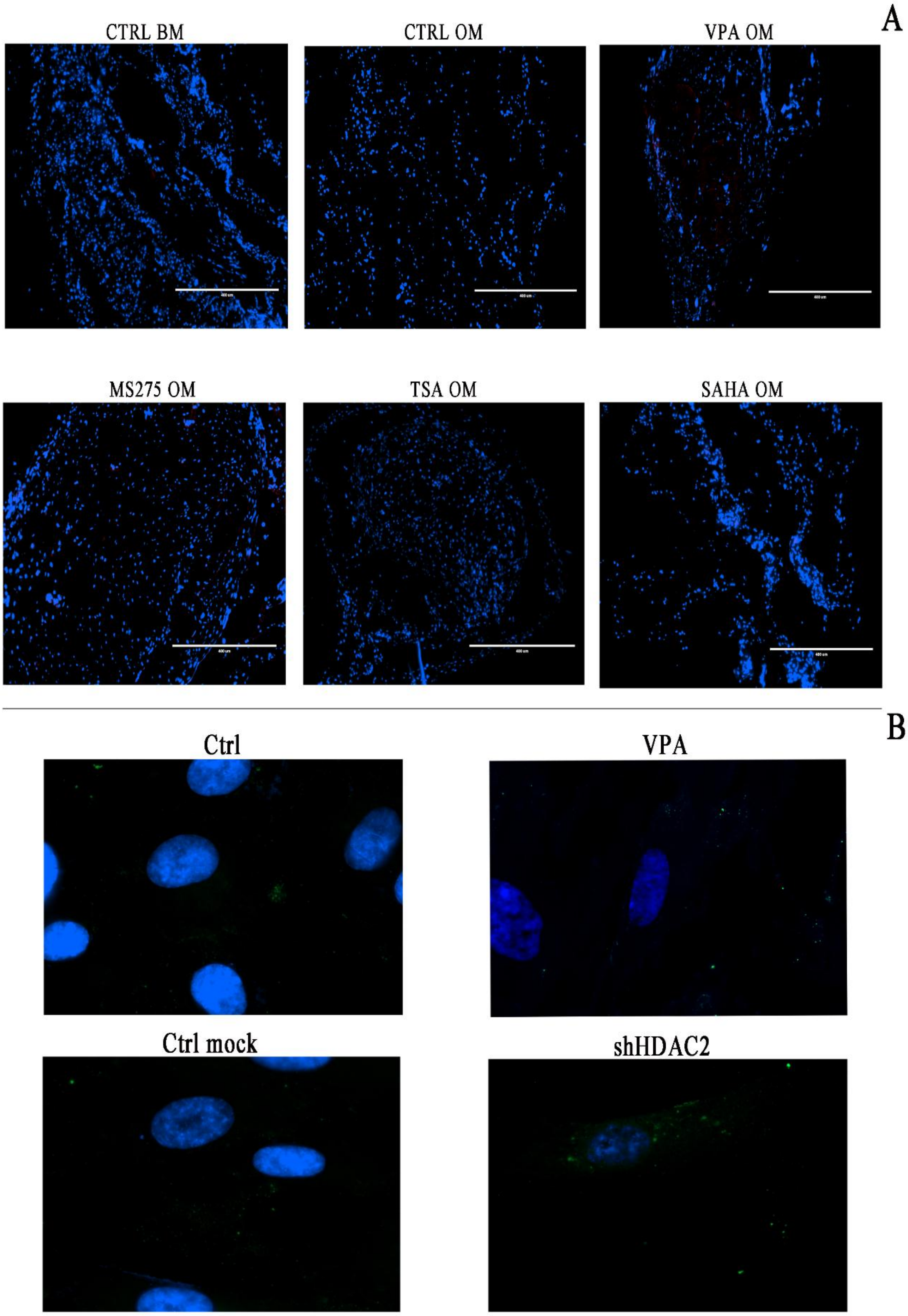

Figure 4. Background controls of the fluorescent images. Background control of in vivo immunofluorescence (A). Background control of GR immunofluorescence (B).

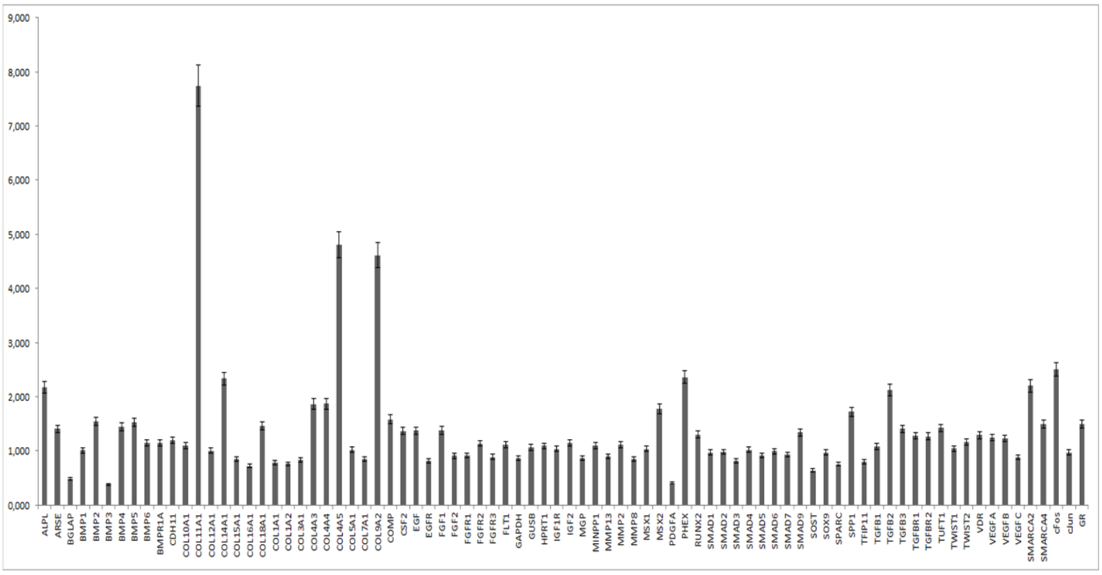

Figure 5. Gene expression analysis. Evaluation of gene expression analysis after VPA treatment.
